# Genome Editing Tools in Plants

**DOI:** 10.3390/genes8120399

**Published:** 2017-12-19

**Authors:** Tapan Kumar Mohanta, Tufail Bashir, Abeer Hashem, Elsayed Fathi Abd_Allah, Hanhong Bae

**Affiliations:** 1 Department of Biotechnology, Yeungnam University, Gyeongsan 38541, Korea; tufail.arab@gmail.com; 2 Botany and Microbiology Department, College of Science, King Saud University, Riyadh 11451, Saudi Arabia; habeer@ksu.edu.sa; 3 Mycology and Plant Disease Survey Department, Plant Pathology Research Institute, Agriculture Research Center, Giza 12619, Egypt; 4 Plant Production Department, College of Food and Agriculture Science, King Saud University, Riyadh 11451, Saudi Arabia; eabdallah@ksu.edu.sa

**Keywords:** genome editing, homologous recombination, Zinc finger nuclease, TALEN, pentatricopeptide repeat protein, CRISPR/Cas9, adenine base editors, RNAi, site-directed sequence editing, oligonucleotide-directed mutagenesis, cisgenesis and intragenesis, plastid genome, synthetic genomics

## Abstract

Genome editing tools have the potential to change the genomic architecture of a genome at precise locations, with desired accuracy. These tools have been efficiently used for trait discovery and for the generation of plants with high crop yields and resistance to biotic and abiotic stresses. Due to complex genomic architecture, it is challenging to edit all of the genes/genomes using a particular genome editing tool. Therefore, to overcome this challenging task, several genome editing tools have been developed to facilitate efficient genome editing. Some of the major genome editing tools used to edit plant genomes are: Homologous recombination (HR), zinc finger nucleases (ZFNs), transcription activator-like effector nucleases (TALENs), pentatricopeptide repeat proteins (PPRs), the CRISPR/Cas9 system, RNA interference (RNAi), cisgenesis, and intragenesis. In addition, site-directed sequence editing and oligonucleotide-directed mutagenesis have the potential to edit the genome at the single-nucleotide level. Recently, adenine base editors (ABEs) have been developed to mutate A-T base pairs to G-C base pairs. ABEs use deoxyadeninedeaminase (TadA) with catalytically impaired Cas9 nickase to mutate A-T base pairs to G-C base pairs.

## 1. Introduction

From the beginning of plant domestication approximately 10,000 years ago, conventional plant breeding methods were the most successful approach for developing new crop varieties. Conventional plant breeding has contributed enormously towards feeding the world and has played crucial roles in the development of modern society. Pre-genomic breeding programs have led to the development of stress-tolerant and high-yielding crop varieties. Breeding programs of the past century have relied on natural and mutant-induced genetic variations to select for favorable genetic combinations. The traditional breeding program that is conducted by mutagenesis using chemical compounds or irradiation, followed by screening for desired mutations, has several drawbacks. Methods using mutagenesis, intergeneric crosses, and translocation breeding are non-specific; and sometimes large parts of the genome are transferred instead of a single gene, or sometimes thousands of nucleotides are mutated instead of a single nucleotide. Therefore, transgenic breeding programs surfaced towards the end of the 20th century (1990) to overcome such problems. The 21st century is regarded as the post-genomic era and, the availability of genome sequence data for multiple crop plants has revolutionized plant breeding programs. Whole-genome sequencing, transcriptome sequencing, identification of small nucleotide polymorphisms (SNPs) and other molecular markers such as Random Amplified Polymorphic DNA (RAPD), Restriction Fragment Length Polymorphism (RFLP), Amplified fragment length polymorphism (AFLP), and Single Sequence Repeats (SSR) have made it possible to create comprehensive genetic and lineage maps to determine potential quantitative trait loci (QTLs) of agronomic importance [1]. Genomics is rapidly gaining importance in molecular breeding programs. Combinations of genomic tools with conventional breeding techniques have opened new doors in genome-based breeding programs. In addition, transcriptomic studies using next-generation sequencing, microarrays and other methods in combination with QTL analyses have led to the development of expression QTLs (eQTLs), which enable the discovery of multiple QTLs simultaneously [1]. Further, a systems biology approach in combination with molecular markers, QTLs, eQTLs, lineage maps and sequence data has been very useful for the identification of agronomic traits [1]. However, novel and desired agronomic traits need to be integrated into the appropriate crop plants in order to maximize benefits. The implementation of comprehensive synthetic biology tools, which are popularly known as “genome editing tools” [2], is required to carry out the task of integrating desired traits into crop genomes. Synthetic biology uses the rational design of biological molecules to achieve a desired goal. Synthetic biological tools act with precision, accuracy, and predictability, and do away with the messiness of inaccuracy. The use of synthetic biology requires a complete understanding of the biological processes that need to be integrated into the genome. Several DNA, RNA, and protein-based tools have been developed to edit and incorporate suitable agronomic traits into the desired crops. Here, we have tried to provide an overview of all the existing genome editing tools and their potential applications. 

Random integration of genes into the existing genomes of target organisms to obtain a transgene construct is one of the most common mechanisms for gene targeting (GT) [3]. The gene targeting approach, which was used for the first time in mouse embryonic stem cells, failed to deliver similar results in plants [4] because embryonic stem cells are more efficient at homologous recombination than other cells [5]. Hence, plant biologists used transposons or retro-transposons to incorporate a transfer DNA (T-DNA) insertion mutant, resulting in random insertions [6]. Sometimes, the random insertion fails to completely knockout the open reading frame (ORF) of a gene, leading to the increased possibility of obtaining mutant plants with partial functions, dominant-negative effects, or aberrant protein products. The introduction of single nucleotides into the genes (or amino acids into the proteins) cannot be completed using such methods. Hence, chemical mutagenesis methods and target-induced local lesions in genomes (TILLING) have been developed to overcome such problems [7,8,9]. However, these techniques have led to off-target mutations in addition to the mutations of interest. Therefore, novel technologies are needed to overcome such problems in plant-genome editing. In this manuscript, we provide detail about major genome editing tools that can be used for novel trait discovery in plants. In addition, we describe details about the available genome editing tools, and how these tools can be improved for better application.

## 2. Homologous Recombination

**Figure 1:**
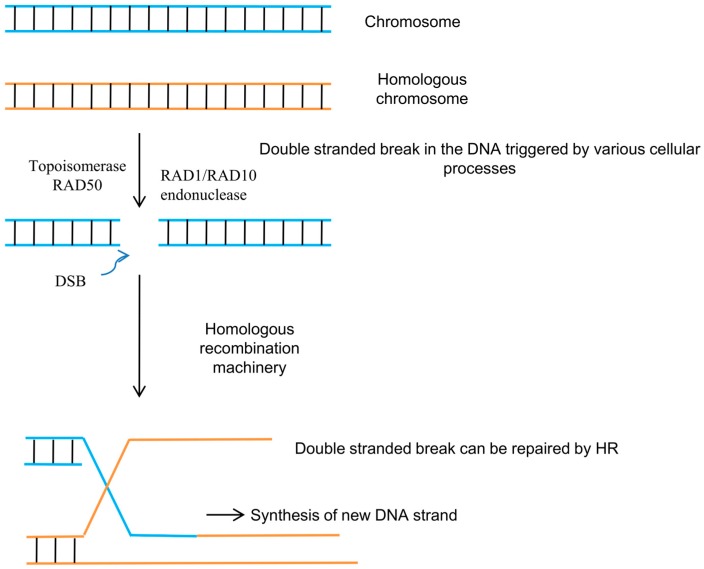
Homologous recombination induced repair of double stranded breaks (DBS). HR: homologous recombination.

Chromosomal recombination (homologous recombination: HR) is the natural and most efficient genome engineering system that is present within the cell [10]. Therefore, a similar mechanism with a minor or no error rate can be useful in genome editing technologies. This method can be used for genome editing by the initiation of double-stranded breaks (DSB) in the chromosome (Figure 1) [11,12]. DSBs lead to meiotic recombination during cell division. DSBs are highly conserved in eukaryotes and can be initiated at specific sites, thus providing a great platform for gene targeting. The HR initiated in a specific region is known as a recombination hotspot. Recombination hotspots are targeted by the Spo11 complex to form a DSB (Figure 1) [13]. The Spo11 protein (DNA topoisomerase) acts in combination with Mre11 (double-strand break repair protein), Rad50 (DNA repair protein), and NBS1 (meiotic recombination protein) [13]. The DSB that is induced by endonuclease leads to the generation of single-stranded 3′ overhangs. The double-stranded ends generated by the DSB are protected from degradation by binding with the Ku heterodimer [14,15,16]. If the DSB generates 3′ overhangs, it is trimmed down; single-stranded regions are replete with repair synthesis. In synthesis-dependent strand annealing (SDSA), a single-stranded 3′ end penetrates into a homologous double strand and forms a D-loop-like structure. Post-elongation, the strand is displaced from the D-loop-like structure and anneals with the 3′-homologous strand. DNA helicases AtFANCM and AtRECQ4A and nuclease AtMUS81 play important roles in SDSA [17,18]. If the DSB occurs within the ORF of a gene, it might result in a frameshift, which might lead to the knockdown of the gene function. The DSB triggers homologous recombination between the two chromosomes (Figure 1). The broken chromosome ends yield single-stranded DNA (ssDNA) tails that target the homologous chromosome to pass the genetic information to the donor chromosome [13]. After the exchange of genetic material with the donor chromosome, the double-stranded break is ligated by the double-stranded break repair enzyme [13]. If the DSB occurs between two closely repeated DNA sequences, then the annealing can be performed by a single-strand annealing (SSA) process [13]. SSA is much more important for those genomic portions that contain tandem repeats. The RAD1/RAD10 endonuclease is involved in cleavage of the complementary strand before ligation by SSA [19]. Non-homologous end joining (NHEJ) is the principal mechanism of DSB repair in plant cells [14]. NHEJ is mediated by two mechanisms, namely, classical NHEJ (cNHEJ) and alternative NHEJ (aNHEJ) [20]. In addition to cNHEJ and aNHEJ, there may be other alternative end-joining mechanisms [21]. However, due to the repetitive nature of plant genomes, the gene-targeting rates that are using homologous recombination are very low in higher plants and range from 10^3^ to 10^−6^ [22,23]. Homology-directed repair (HDR) can also facilitate the generation of targeted mutations at specific genomic locations using the sequences of donor DNA [24]. In such cases, to obtain the desired sequence modification, donor HDR DNA contain an approximately 750–1000 kb long homologous sequence that flanks the genomic cleavage site [24]. However, the frequencies of HDR in somatic cells are very low, and, hence, the number of genomic modifications that is obtained using HDR techniques is very low. 

## 3. Zinc Finger Nucleases

**Figure 2:**
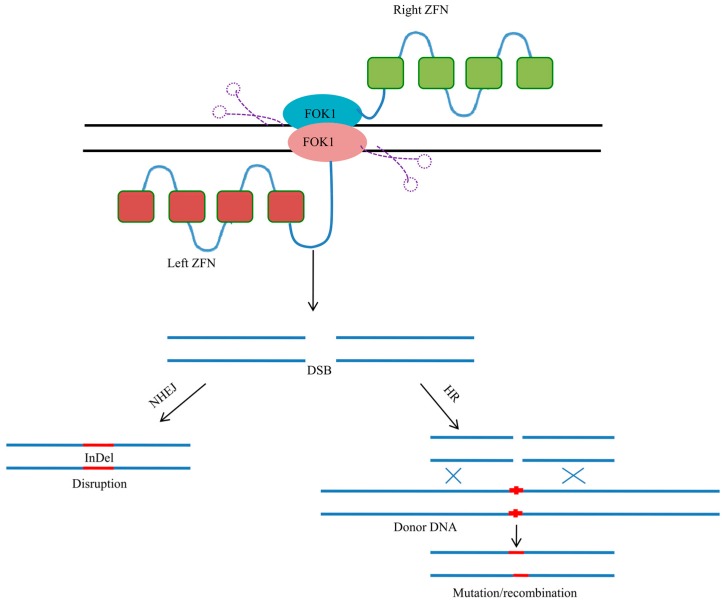
Schematic representation of Zinc finger nucleases (ZFNs). ZFNs constitute FOK1 nuclease domain at the carboxyl end and a Zinc finger protein at the amino terminus. FOK1 nuclease creates double stranded breaks (DSB), which repaired by nonhomologous end joining (NHEJ) or homologous recombination (HR) to bring desired mutation/recombination of gene.

Zinc finger nucleases (ZFNs) are custom-designed, targetable DNA cleavage proteins that are designed to cut DNA sequences at specific sites [25]. ZFNs facilitate targeted gene editing through the creation of DSBs in DNA to replace the gene by homologous recombination (Figure 2). Each ZFN contains a DNA-binding domain with a chain of two-finger modules, which recognizes a unique 6-bp hexamer in the DNA sequence, and a DNA-cleaving domain consisting of a FokI nuclease domain (Figure 2) [26,27]. These domains are joined together to form a zinc finger protein (ZFP). When the DNA-binding and DNA-cleaving domains are fused together, they form a highly specific genomic scissor [26,27]. ZNF-mediated gene targeting introduces site-specific double-stranded breaks into the DNA sequence and permanently edits the genome via the ligation of DSBs [26,27]. The most important factor for the application of ZFN-based genome editing is the dependence of this technique on the generation of ZFPs that can precisely target a specific DNA sequence in the genome. The Cys2His2 ZFP provides the best possible structure for developing suitable ZFNs with the required sequence specificities [28]. This ZFP consists of approximately 30 amino acids and has a ββα structure that is stabilized by the chelation of zinc ions to conserved Cys2His2 amino acids [29,30]. The ZF motif binds to the DNA sequence in the genome by incorporating its α-helix into the major groove of the DNA double helix [30]. The amino acids at positions −1, +1, +2, +3, +4, +5, and +6 of the α-helix of the zinc finger are responsible for sequence-specific interactions of ZFN with the DNA sequence [30,31,32]. Each of the fingers binds a triplet sequence of the DNA. Binding to a longer DNA strand is made possible by linking multiple zinc finger motifs to form ZFPs [33,34,35]. The methylase domain (M), FokI-cleavage domain (N), transcription activator domain (A), and transcription repressor domain (R) are fused with ZFP to form a ZFN [36,37,38,39].

The engineering of ZFNs that recognize and cleave specific target sequences largely depends on the reliable engineering of ZFPs that can recognize the target of interest. As ZFNs specifically bind to triplet DNA, the presence of 64 triplet variants in the genome makes it challenging to design ZFNs that bind each and every triplet variant. However, ZFNs that bind 5′-GNN-3′, 5′-ANN-3′, 5′-CNN-3′, and 5′-TNN-3′ have been experimentally validated [40,41,42,43,44]. The presence of an Asp residue at the 2nd position of the α-helix in the preceding ZF motif promotes cross-strand contact outside the triplet codon, which results in an overlap of target sites [30,45]. Therefore, when the Asp residue is present at the second position of the preceding α-helix of the ZFP, it binds to a four-base-pair DNA target sequence instead of a triplet codon, thus complicating the design strategy. However, in many instances, the ZF motifs of ZFPs make sequence-specific contacts with only two nucleotides of the triplet DNA [30,46], and the presence of an Asp residue at the 2nd position increases the affinity and the specificity of the ZF motif for triplet sub-sites. In the absence of an Asp residue at the 2nd position, only two bases of the triplet DNA are recognized, resulting in the possibility of the recognition of degenerate sites [47]. A set of three ZFPs recognizes an 18 bp target sequence; depending upon the specificity of each ZF to its corresponding triplet, the actual recognition site may vary between 12 and 18 bp [47].

Once a particular ZFP recognizes a specific nucleotide site, the FokI restriction endonuclease comes into play. FokI is a type II restriction enzyme that recognizes the non-palindromic pentadeoxyribonucleotide sequence 5′-GGATG-3′:5′-CATCC-3′ in double-stranded DNA and cleaves the DNA 9/13 nucleotides downstream of the recognition site. Upon binding of the DNA-binding domain of FokI to the recognition site, a signal is transmitted to the endonuclease domain, and cleavage occurs. ZFNs dimerize the nuclease domain to carry out double-stranded cleavage of DNA [48]. Three ZFNs require six base pair recognition sites to dimerize the nuclease domain, leading to the generation of a DSB. Effectively, ZFNs have 18-base-pair recognition sites, which are sufficient for the recognition of a unique DNA sequence [47]. Two ZFNs with contrasting sequence specificity and recognition sites can work as a heterodimer to produce the DSB.

After the generation of a DSB (Figure 2), it is necessary to target a suitable gene in the genome. A gene with the consensus sequence 5′-GNNGNNGNN-3′ can be targeted by a simple assembly approach [49]. It is also possible to target sequences with mixtures of ANN, GNN, and CNN triplets [40,42,43]. The gene fragment can be either transferred to a homologous segment of the genome or targeted randomly. In cases of random integration, the DSB is ligated by non-homologous end joining, or can be ligated through homologous recombination otherwise (Figure 2). Using ZFNs, we can rapidly and randomly disrupt or integrate any genomic loci in the genome. Mutations that are made through ZFNs are permanent and can be heritable. The selection of a ZNF strategy can be conducted by a bacterial two-hybrid system that uses ZFN-DNA interactions to activate the *HS3* gene. A bacterial one-hybrid system can also be used to select ZFPs and to analyze sequence specificities in vivo [50]. 

## 4. Transcription Activator-Like Effector Nuclease

**Figure 3:**
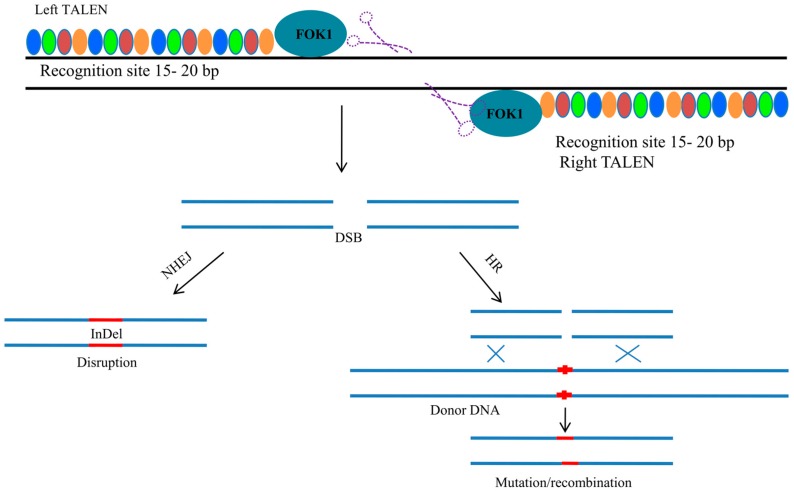
Schematic representation of TALENs (Transcription activator-like effector nucleases) having TALE repeats as shown by colored discs and a FOK1 endonuclease which cleaves the DNA.

Transcription activator-like effectors nucleases (TALENs) have emerged as an alternative to ZFNs as tools for effective genome editing in plants (Figure 3) [51]. In principle, TALENs uses DSBs in a manner similar to ZFNs (Figure 3). TALENs are similar to ZFNs that contain non-specific FokI endonucleases. However, the FokI domains of ZFNs fuse with specific DNA-binding domains of highly conserved repeats derived from transcription activator-like effectors (TALEs) [51]. TALE proteins are found in *Xanthomonas* bacteria, which secrete TALEs to alter gene transcription in host plants [52,53]. The DNA-binding domains of TALEs contain up to 30 copies of 33–34 amino acid sequences that are highly conserved, except for 12th and 13th positions. The 12th and 13th positions are called the repeat-variable diresidue (RVD), and exhibit substantial correlation with specific nucleotide recognition. Each repeat can recognize a single base, and hence, new binding sites can be assembled for any DNA sequence. The FokI domain functions as a dimer, where the non-specific DNA cleavage domain of the FokI endonuclease can be used to design a hybrid nuclease [48,54,55]. The number of amino acid residues between the DNA-binding domain and the FokI cleavage domain and the number of bases between two separate TALEN-binding sites are important parameters that are affecting the activities of TALENs. The recognition of amino acids and DNA-binding TALE domains requires effective engineering of proteins. Upon the construction of TALENs, they are transferred to the plasmid vector and are then transformed into the target cells. Later, the gene product is expressed and enters the nucleus, where it carries out the necessary editing of the genome. Alternatively, the TALEN construct can be transformed into cells as mRNA, which eliminates the possibility of genomic integration of the TALEN. The mRNA-based approach increases the possibilities of homology-based repair (HDR) and leads to successful gene editing. The TALEN technology has been successfully used in *Oryza sativa* [56]. The promoter region of the bacterial blight susceptible gene *Os11N3* was targeted using TALEN technology, which led to the generation of disease-resistant rice [56]. A comparative study between ZFNs and TALENs to target particular genes showed the highest cleavage rates for the TALENs. This result was due to the ease of design and high cleavage activities of TALENs and the limitless range of targets that can be acted upon by TALENs [51]. TALEN technology has been efficiently used to generate knockout mutants of *Arabidopsis thaliana* [57]. Although TALEN technology is very efficient and is superior to ZFNs, the construction of engineered TALE repeats is challenging because it requires multiple identical repeat sequences [51].

## 5. Pentatricopeptide Repeat Proteins

**Figure 4:**
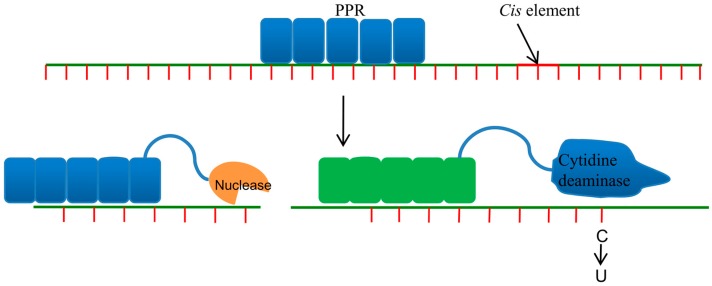
RNA editing by pentatricopeptide repeat proteins (PPR). PPR proteins with a nuclease or a cytidine deaminase domain trigger RNA modifications as shown.

Organellar genomes possess relatively low numbers of promoters, and the half-life periods of organellar RNA are relatively higher than those of nuclear RNA. Therefore, transcriptional regulation (RNA editing, RNA cleavage, RNA splicing, and translation) is insufficient for controlling gene expression in organellar genomes, and, hence, organelles have developed a great array of RNA-binding proteins to regulate gene expression at the post-transcriptional level [58,59]. The pentatricopeptide repeat protein (PPR), which is found in the organellar genome, is a mediator protein that regulates post-transcriptional regulation. PPRs are traditionally characterized by the presence of 35-amino-acid tandemly repeated motifs [59]. Depending upon the number of amino acids, PPRs are classified as P-class (35 amino acids), L-class (35–36 amino acids), S-class (short, approximately 31 amino acids), and E-class (extended domain) [60,61,62]. The PLS-type PPRs contain a highly conserved DYW (Aspartate-Tyrosine-Tryptophan) tripeptide motif at the C-terminal end that most likely acts as an editing domain [63]. The DYW domain possesses zinc-binding affinity, which is essential for catalysis and hence for editing [64]. The L-class PPRs contain amino acid residues that are possibly involved in RNA binding corresponding to the targeted nucleotide. The extended E-domain PPR is not catalytic, but it helps in protein-protein interactions with the editing enzyme [61,62]. PPRs contain up to 30 tandem repeats and consist of approximately 35 amino acids [59]. The α-helices form an α-solenoid anti-parallel helix-turn-helix structure that provides the basis for sequence-specific RNA binding. Barkan et al. (2012) reported that positions 6 and 1 (position 4 and 34 in the Pfam model) of the PPR motif is responsible for nucleotide recognition and RNA binding (Figure 4) [65]. At positions 6 and 1, PPR motifs recognize their cognate target transcripts in a modular fashion. The amino acid residues at positions 6 and 1 determine the nucleotide to which the PPR will bind, and each PPR motif only binds one nucleotide [66]. PPRs bind to the 5′ ends of RNA in a parallel fashion. The interactions between PPR motifs and nucleotides occurs via van der Waals interactions, and a threonine at position 6 in combination with asparagine at position 1 recognizes adenine nucleotides, whereas asparagine and aspartic acid at positions 6 and 1, respectively, recognize uracil nucleotides (Figure 4) [65,67,68]. PPRs can directly bind to specific RNA sequences as monomers by employing the amino acid residues at positions 6 and 1 position. The amino acid at position 3 helps in the interaction of PPRs with tRNA, and this position is usually occupied by a hydrophobic amino acid [69]. Due to higher diversity in the amino acid composition of PPR motifs, these motifs provide greater versatility for engineering. The LAGLIDADG motif involved in splicing events is also found in PPRs [70,71]. The Small MutS-related (SMR) group of proteins with C-terminal SMR domains resemble PPRs (P-class) that is involved in DNA recombination and repair in bacteria [72,73]. The PPR motifs in combination with SMR domains provide RNA-binding ability and endonucleolytic activity to SMR, leading to RNA cleavage [74]. 

The wide diversity of natural motifs in PPRs provides an excellent basis for gene editing. PPRs bind tRNA in a parallel orientation through a modular recognition mechanism. The maize PPR10 protein that contains a 17 PPR motif, where motifs 6 (N6D1′) and 7 (N6N1′) bind to C and U nucleotides, respectively, in the natural target (Figure 4) [65]. Amino acid substitutions were carried out at specific amino acid residues to modify the predicted RNA-binding specificities to GG (N6D1′), AA (T6N1′), CC (T6S1′), or UU (N6N1′) [65,68]. In vitro analysis revealed changes in the binding specificities of these variants. Excellent specificities were observed for the GG and AA variants, whereas the CC and UU variants had poorer specificities [65]. However, the prediction of natural binding sites and off-target binding sites of engineered PPRs remains challenging because the amino acids at positions 6 and 1′ can lead to degenerate codes, and less than two-third of the naturally occurring combinations can be translated simultaneously. Additionally, understanding the energetic parameters requires the establishment of meaningful RNA-PPR interactions, and the energy costs of mismatches at different positions in an RNA-PPR duplex imply that accurate predictions are needed in order to predict potential binding sites [65]. The prediction of binding sites is further complicated by the presence of gaps in a PPR-RNA duplex. The PPR HCF-152 and CRP1 contain a gap in their predicted PPR-RNA duplex with the non-contiguous segment of either protein (HCF152) or RNA (PPR10) [65]. The alignment of cognate PPR-RNA binding contains contiguous duplexes of nine motifs and eight nucleotides. However, there is no alignment gap between L-class PPRs and RNA [65]. The amino acids at position 6 differ between the P- and S-versus L-type PPR motifs. Thus, it can be easily speculated that the L-motif does not bind to any nucleotide bases and allows a mini-gap in every third nucleotide.

## 6. CRISPR/Cas9

**Figure 5:**
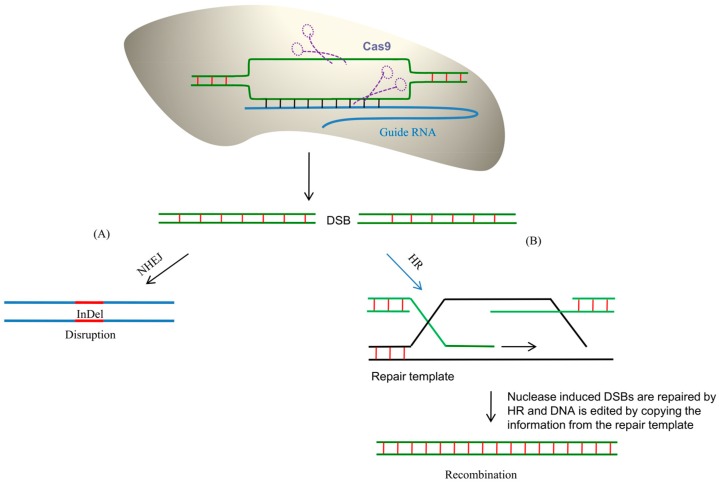
Genome editing mechanism. A double stranded break is generated by a nuclease, such as Cas9 in the genome and DSB produced can be repaired in two ways. (**A**) Non-homologous end joining can also repair the DSBs by making small insertions and deletions. (**B**) HR driven repair mechanism can correct the DSBs from the template donor plasmid which has modifications to be introduced in the genome.

Clustered regularly interspaced short palindromic repeats (CRISPR)/Cas is a family of DNA sequences that is commonly found in bacteria. It contains fragments of DNA from viruses that have attacked the bacterium. These DNA fragments are used by the bacterium to recognize and destroy DNA from further attacks, and thereby protect themselves. CRISPR/Cas acts as a typical bacterial immune system that provides the bacteria with resistance to foreign genetic material. The CRISPR system comprises CRISPR RNA (crRNA), trans-activating CRISPR RNA (tracrRNA), the Cas9 nuclease, and the protospacer adjacent motif (PAM) (Figure 5). Naturally occurring CRISPR systems integrate the foreign DNA sequence into the CRISPR cluster [75]. Then, the CRISPR cluster that is harboring the foreign DNA produces crRNA (approximately 40 nt long) containing the PAM region, which is complementary to the foreign DNA site. The crRNA hybridizes with the tracrRNA to form a guide RNA (gRNA) (Figure 5). The gRNA activates the Cas9 system and binds to Cas9. Twenty nucleotides at the 5′ end of the gRNA direct the Cas9 nuclease to the complementary base pair with the targeted DNA, leading to RNA-DNA complementary base-pairing [75]. The prerequisite for cleavage is the presence of a PAM motif downstream of the target DNA; the PAM motif usually contains 5′-NGG-3′ or 5′-NAG-3′ [76,77]. Specificity is provided by the “seed sequence”, which is present approximately 12 nucleotides upstream of the PAM motif and which should match between the RNA and target DNA [78]. Using this procedure, Cas9 nuclease activity can be directed to any DNA sequence [75]. The Cas9 system induces DSBs, which are subsequently ligated by NHEJ or HDR [75] (Figure 5). Some Cas9 variants cleave only at one site (nickase) of either the complementary or the non-complementary strands of the target DNA. The Cas9 nickase induces HDR with reduced levels of NHEJ indels [79,80]. By using one Cas9 nuclease and multiple gRNA, more than one site can be targeted and altered simultaneously [81]. This process is very useful when one gRNA is inefficient at disrupting a targeted gene or when altering more than one gene at the same time.

One of the major criticisms regarding the usefulness and specificity of the CRISPR/Cas9 technology is the relatively high frequencies of off-target mutations [77,78]. However, the off-target mutations are rare in plants. Only 1.6% off-target effects were predicted in rice [82]. The mismatch was confined to position 11, which is present upstream of the PAM motif. However, no off-target mutations were observed in *A. thaliana*, *Nicotiana benthamiana*, *Triticum aestivum,* and *O. sativa* [83,84,85,86,87,88,89].

Previously, it was considered that the 20 nt gRNA sequence determines the specificity; however, it later found that only 8–12 nt at the 3′ end (the seed sequence) is required for recognition of target sites [79,90,91], and multiple mismatches towards the PAM motif can be tolerated, depending upon the arrangement of the PAM motif [77,92,93]. DNA sequences that contain a missing base (gRNA bulge) or an extra base (DNA bulge) at various positions in the corresponding gRNA sequences induce off-target cleavage [94]. The specificity of CRISPR/Cas9 at non-seed positions in the crRNA spacer possess the intrinsic property that reduces the possibilities of point mutations [95]. Appropriate gRNA design can greatly facilitate the reduction of off-target editing of the genome. Due to the Watson-Crick base-pairing of CRISPR/Cas9 with its target sequence, off-target sites can be easily predicted by using sequence analysis [96]. The CRISPR/Cas9 system can be reprogrammed to test off-target effects rapidly and in a cost-effective manner. In regard to off-target cleavage, the mismatches are preferably tolerated at the 5′ end of the gRNA when compared to the 3′ end [75]. However, some mismatches at the 5′ end can have a significant effect, whereas 3′ ends do not affect Cas9 activity [93]. In 6 × 10^9^ random DNA bases, the 20-nt protospacer could have hundreds to thousands of potential off-target sites [75]. Higher GC content of the gRNA: DNA hybrid stabilizes the binding of gRNA to the DNA, and hence, the possibility of off-target mutations is very low. GC content below 30% leads to high rates of off-target mutagenesis [92,93]. Reducing the concentration of gRNA and Cas9 can reduce off-target mutations [75]; however, in a few cases, reduced mutation of the target site was also observed [75,93]. Modified gRNA with truncated 3′ ends (within tracr-derived sequences) or with two guanine nucleotides appended to the 5′ end (before the complementary region) lead to better on-target to off-target mutation ratios [75] with reduced genome editing efficiencies [75]. Using a paired nickase strategy, off-target nicks can be generated at the target site using gRNA and Cas9 nickase. Paired Cas9 nickase targets sites separated by 4 to 100 bpon the opposite strand of the DNA and is capable of inducing indel mutations and HDR with single-strand DNA oligonucleotide donors [75]. The addition of a second gRNA and the replacement of Cas9 nuclease with Cas9 nickase leads to lower levels of off-target mutations [75]. A single monomeric Cas9 nickase can induce indel mutations in genomic loci [80,97,98,99]. However, the type of gRNA used is one of the most crucial factors for achieving high efficiencies in genome editing. Longer single gRNA, harboring variable lengths of tracrRNA sequences towards the 3′ end, result in higher editing frequencies than shorter gRNA [77]. Fu et al. (2014) reported that off-target effects can be substantially reduced by the use of short gRNA sequences that are truncated at the 5′ ends [98]. The truncated gRNA shares 17–18 complementary nucleotides, and shows lower mutagenic effects off-target sites with high sensitivity to mismatches at the gRNA: DNA interface [98]. However, longer single gRNA with more tracrRNA towards the 3′ ends yield higher editing frequencies when compared to shorter gRNA sequences [77]. The most frequently used single gRNA design possesses approximately 100 nucleotides [75]. The role of the promoter used to express the gRNA is of particular interest as it can limit the options of target sites. The UBI, U3, U6, and UBQ promoters show high mutation frequencies when compared to other promoters [75,100,101]. 

To further advance the CRISPR/Cas9 genome editing system, given a particular gene and species, computational models can precisely design suitable gRNA for genome editing and can predict off-target sites. An unbiased method for the detection of genome-wide off-target sites is used for the in-silico prediction of off-target sites [102]. The spCas9 PAM-variant D1135E contains a single point mutation that increases the specificity for the 5′-NGG PAM [103]. This variant significantly decreased editing at the 5′-NAG and 5′-NGA PAMs, and improved genome-wide specificity [103]. Similarly, spCas9-HF, with four mutations, weakens the binding of Cas9 to target DNA and enhances the stringency of gRNA:DNA complementation for Cas9 activation. Off-target editing was almost completely avoided using this variation [104]. Engineered eSpCas9, with three mutations within the nucleotide-groove, the off-target sites of Cas9 increases the stringency of gRNA for nuclease activation [105].

In addition to genome editing, the CRISPR/Cas9 system can be used for the ectopic regulation of gene expression. Through the regulation of gene expression, we can understand the function of a gene, and can also engineer novel genetic regulatory circuits for synthetic biology [78]. The regulation of gene expression is mediated by inducible or repressible promoters, and disabled nucleases can be used to regulate gene expression [78]. The catalytically inactive/dead Cas9 (dCas9) is unable to cut DNA but can bind to target sites through gRNA. Expression of dead Cas9 as a fusion protein with the activation or repression domains of transcription factors can lead to reversible transcriptional control of target genes [106,107]. The fusion of dCas9 with the C-terminal EDLL domain of PDS, which contains the TAL activation domain, leads to the generation of transcriptional activators. Similarly, fusion of dCas9 with the SRDX domain of the ERF transcription factor generates a repressor [78]. The transcriptional activities are influenced by the position of gRNA with respect to the transcription start site [78]. The naked dCas9, without the effector domain, represses synthetic and endogenous genes by inhibition of transcription initiation and elongation. The dCas9 system can be applied to species that lack a controllable expression system. Multiple gRNA can be targeted to the same or different promoter in order to have transcriptional control of gene expression [108,109,110]. Orthogonal Cas9 proteins mediate simultaneous and independent targeted gene editing and regulation in the same cell [111]. dCas9 can be used to deliver specific cargo to the targeted genomic region [78]. dCas9 that is fused with fluorescent proteins can be used to visualize specific genomic loci of living cells, from which we can learn about chromosome structure and dynamics [112]. A similar approach can be utilized to target histone modifications and DNA methylation, and for the editing of epigenomes [113].

## 7. Adenine Base Editor

**Figure 6:**
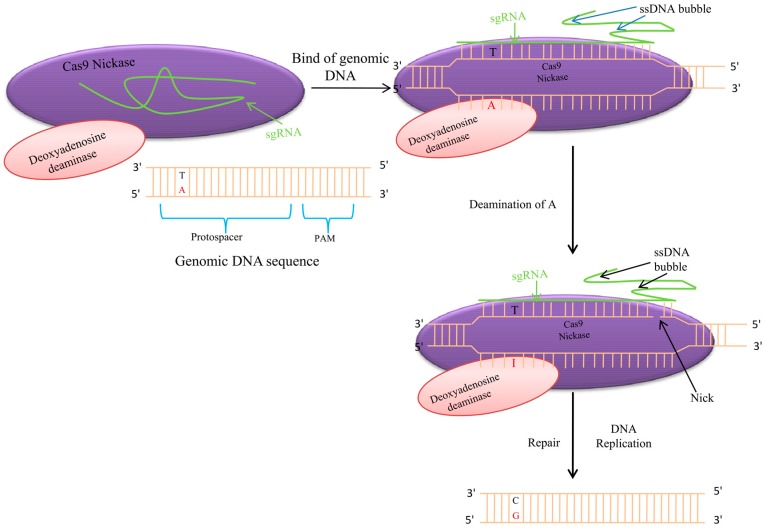
Adenine Base Editor (ABE). ABE mediates conversion of A-T to G-C base pairing using deoxyadenosine deaminase, catalytically impaired Cas9 nickase and a guide RNA (sgRNA). Deamination of adenine is done by deaminase by converting it to inosine (I). During polymerization, polymerase reads I as guanosine (G) and subsequently A-T is replaced by G-C. ssDNA: single stranded DNA

Spontaneous conversion of cytosine and 5-methylcytosine to uracil and thymine, respectively, occurs through hydrolytic deamination in cells and results in C-G to T-A mutations [114,115,116]. This conversion occurs 100–500 times per cell per day [117]. However, these editing processes are confined to only conversion of C-G to T-A. Thus, the process that converts A-T base pairs to G-C base pairs in target genomic loci is the basis for the generation of small nucleotide polymorphisms (SNPs). Adenine base editors (ABEs) can potentially overcome this hurdle (Figure 6). ABEs convert A-T to G-C in bacteria and humans (Figure 6), and are yet to be implemented in plants [117]. Seventh generation ABEs have greatly advanced the conversion of A-T to G-C at targeted genomic loci with very high degrees of accuracy and purity [117]. ABEs use tRNA adenosine deaminase fused to catalytically impaired CRISPR-Cas9 to convert A-T to G-C (Figure 6) [117]. ABEs generate point mutations more efficiently than Cas9 nuclease-based genome editing methods, with high product purity (≥99.9%) and significantly low rates of indels (≤0.1%), and with less off-target mutations [117]. In this process, the deoxyadenosine deaminase TadA is fused with catalytically impaired Cas9 nickase with a corresponding single guide RNA (sgRNA) to perform the task (Figure 6) [117]. Fused TadA and Cas9 bind the target DNA sequence/nucleotide in a guide RNA-programmed manner, thus exposing a small single-stranded DNA bubble. The TadA deoxyadenosine deaminase converts adenine (A) to inosine (I) within the bubble. The I nucleotide pairs, with C, and, during DNA replication, the polymerase reads it as a G nucleotide; hence, A nucleotides are replaced by G nucleotides. Fusion of TadA to the C-terminal end of Cas9-nickase instead of the N-terminal end abolishes the editing activity [117]. Doubling the length of the linker between TadA and Cas9 nickase by using the 32-amino-acid-long (SGGS)_2_-STEN-(SGGS)_2_ linker leads to higher editing efficiencies [117]. TadA is natively found as a homodimer, where one monomer catalyzes the deamination process and the other acts as a docking station for the tRNA substrate [117]. Inactivated N-terminal TadA shows editing potential when compared to the inactivated internal TadA subunit, confirming that the internal TadA subunit is responsible for ABE deamination activity [117]. ABE showed limited editing efficiencies for a target sequence containing multiple adenine nucleotides. However, this problem was overcome when Tad-Cas9 was targeted to two separate sites, namely, TAT and TAA, in the kanamycin resistance gene. The C nucleotide of the UAC triplet anti-codon loop of the tRNA substrate contributes significantly to the enhancement of editing efficiencies. A sequence with alternating nucleotides, namely,5′-A-N-A-N-A-N-A-N-A-N-A-N-A-N-3′, was targeted for editing with two sgRNA, such that an A nucleotide would be located at either the 18th (odd) or the 19th (even) positions from positions 2 to 9 of protospacer. The editing result at all of the 19 sites suggested that different ABE variants have different editing efficiencies with based on the PAM domain at positions 21 to 23 [117]. Therefore, the precise editing specificities and the limits of the editing specificities can vary in a target-dependent manner [117]. The base editing activities near adenines within the editing window are statistically dependent, and the average normal linkage disequilibrium (LD) near target adenines increased substantially upon the evolution of ABEs [117]. This finding reflects that early-stage ABEs edit nearby adenines independently, whereas late-stage ABEs edit nearby adenines processively. During the evolutionary process, TadA might have evolved changes in kinetic behavior, leading to the decreased possibility substrate release. This new genome editing technique can be implemented successfully in animal cell lines, and can be effectively used to obtain desirable traits in plants as well.

## 8. RNA Interference

RNA interference (RNAi) is a post-transcriptional gene silencing mechanism that regulates gene expression in different ways [116,118,119]. The RNAi can be due to siRNAs (small interfering RNAs) [120,121], miRNAs (microRNAs) [122], or piRNAs (PIWI-interacting RNAs) [123,124,125]. siRNAs are 20 to 30-nucleotide-long non-coding single-stranded RNA that are produced from double-stranded RNA, and act as guides for DNA cleavage and translational repression. siRNA activity is based on sequence complementarity and leads to the knockdown of gene function [126,127]. The discovery of RNAi by Andrew Fire and Craig Mello earned them a Nobel Prize in 2006, indicating the importance of RNAi technology. Each small RNA forms a complex with an Argonaute-family protein and base pairs with the target gene in a sequence-specific manner [128,129]. The siRNA forms an RNA-induced silencing complex (RISC) that drives the silencing of target mRNA through repression or degradation [130,131,132,133]. In addition, siRNAs can alter the chromatin configuration and the methylation of the siRNA-binding site [134,135,136]. siRNAs can be endogenous or may arise through exogenous sources or viral infections, whereas miRNAs are derived directly from the genome. During functional studies, it has been observed that siRNA duplexes exhibit perfect base-pairing, whereas miRNA duplexes exhibit mismatches due to the presence of an extended terminal D-loop [137,138,139]. Although the origins of siRNAs and miRNAs are different, the processing pathways of both require RISC.

Plant cells produce only a few siRNAs, and the majority of siRNAs are of viral origin. Therefore, it is necessary to deliver the designed siRNA into the cell for the functional study of any gene. However, the presence of cell walls in plant cells makes the delivery of siRNA into the cell difficult. The delivery of siRNA into plant cells has been achieved by expressing hairpin RNA that fold back to generate a double-stranded region. The double-stranded region acts as a substrate recognition site for DCL/Dicer-like enzyme, which later complexes with Argonaute in order to regulate the silencing process [140,141]. Modified viruses are usually used to induce the RNAi, and hence to knockdown genes, in a process that commonly known as virus-induced gene silencing (VIGS) [142,143,144]. VIGS negates the possibility of introducing transgenes into the target genome, and, hence, this process can be useful in such plant species that are recalcitrant to genetic transformation. In contrast to siRNAs, miRNAs are produced from the endogenous genome and have natural origins. Pre-miRNAs with specific hairpin precursors are cleaved by the Dicer enzyme [141]. miRNAs are evolutionary conserved small RNA, which can control the expression of several genes [145,146,147]. Artificial miRNAs can be generated to cleave the targeted transcript [148,149,150]. A small 7-nucleotide complementarity of the siRNA with the target gene can inhibit the expression of the gene. Therefore, the prediction of off-target effects using siRNA is very difficult.

Due to the limited sequence specificity of siRNAs, it is difficult to conduct large-scale screening of the gene expression. A mutation in a gene always leads to irreversible changes, and the functional effect of the mutation is easily predictable. However, RNAi inhibition shows a wide array of effects that depend upon the target gene and the region of the gene. Two sibling plants that are carrying identical RNAi can also exhibit different effects. Inefficacies might arise due to presence of short siRNAs or due to the targeting of genes that are bound by proteins or are masked by secondary structures.

## 9. Site-Directed Sequence Editing

**Figure 7:**
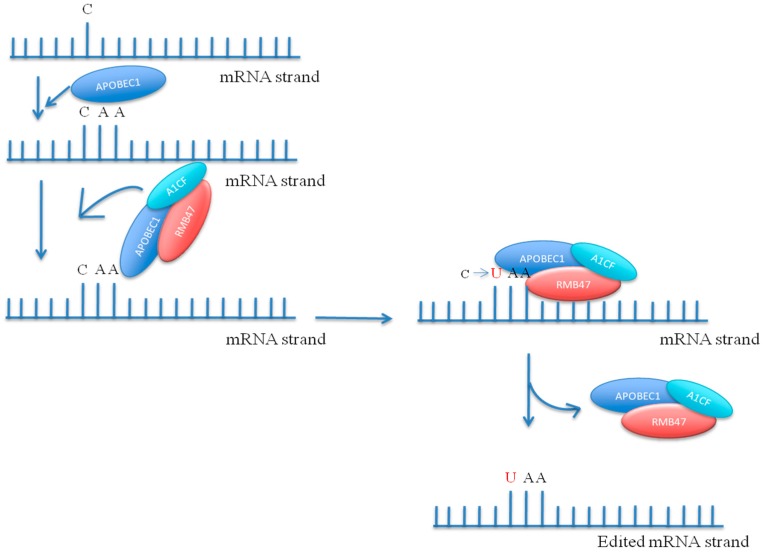
Post-transcriptional modification of C to U by deaminase Apolipoprotein B mRNA editing enzyme, catalytic polypeptide 1 (APOBEC1) in combination with RNA-binding protein A1CF. RMB47 is necessary for APOBEC1 mediated editing.

The pentatricopeptide proteins used in RNA editing harbour C-terminal glutamate (E) amino acid and DYW (aspartate/tyrosine/tryptophan tripeptide) domain [151]. The DYW domain shares homology with protein nucleotide deaminase [152]. If the function of this domain will be confirmed in plants, this editing machinery will resemble the Apolipoprotein B mRNA editing enzyme, (APOBEC1) of mammals that consists of a sequence recognition motif coupled with cytidine deaminase (Figure 7) [153]. The APOBEC1 is a catalytic subunit of an enzyme complex that conducts apolipoprotein (apo) B-mRNA editing [154]. APOBEC1 undergoes dimerization in vitro, and carries out the editing process in presence of complementation factors [154]. The dimerization of APOBEC1 generates an active structure for the deamination of apoB mRNA. Deletion studies on the N- and C-terminal domains showed that the amino-terminal end up to residue A^117^ does not interfere with the dimerization process, whereas deletion at the carboxy-terminal resulted in reduced dimerization [154]. Amino acid clusters, R^15^R^16^R^17^ and R^33^K^34^, are essential for apoB-mediated mRNA editing [154]; mutation of the amino acids at these positions completely abolishes the editing activity in vitro. In addition, the C-terminal region of APOBEC1 contains leucine-rich motifs, and the amino acids at positions 181 to 210 are crucial for apoB-mediated gene editing [154]. Amino acid substitutions at these positions demonstrated that L^182^, I^185^, and L^189^ are important amino acids that are crucial for mRNA editing [154]. ApoB mediates very distinctive RNA editing, wherein a C-to-U modification in the first base of the CAA codon generates a UAA stop codon [154]. The editing of C to U is common in organelles of angiosperms, and editing of U to C is common in ferns and hornworts [155]. However, the enzyme responsible for such editing is not known. Editing of A to I is common in metazoans, and is mediated by deaminase. The enzymatic domain of deaminase coupled with PPR can be very useful for mRNA editing.

## 10. Oligonucleotide-Directed Mutagenesis

**Figure 8:**
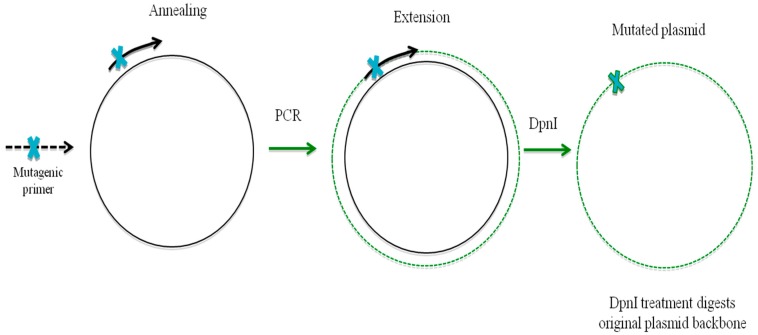
PCR based site directed mutagenesis: Mutated primers complimentary to the plasmid sequence are designed and later PCR based amplification resulting from the extension of the annealed primer completes the synthesis of the new mutated plasmid that harbors a mutation introduced through the primer. DpnI digestion removes the methylated parental non-mutated plasmid sequences, leaving the newly synthesis plasmid for future transformation experiments.

Oligonucleotide-directed mutagenesis (ODM) offers a precise and non-transgenic genome editing technique to manipulate genomic DNA using synthetic oligonucleotides (Figure 8). Using ODM, one to a few nucleotides can be precisely exchanged/introduced into gene of live cells using a plasmid as a template. The synthetic oligonucleotide in this process is a single-stranded sequence that is complementary/homologous to one strand of the duplex DNA, with minor mismatches. The mismatch pairing is corrected by the cellular repair system, which leads to specific mutations. The use of higher concentrations of oligonucleotides in cells leads to site-specific insertions of oligonucleotides into the genome through the action of the cellular DNA repair mechanism [156]. During the site-directed mutagenesis, single-stranded oligonucleotide strands binds with the target DNA and make it three-stranded, which results in the formation of a displacement loop (D-loop). The formation of the D-loop is mediated by the unwinding of chromosomal DNA during replication and transcription. This process leads to an increase in the accessibility of the target site, which initiates the annealing process. Later, sequence transfer to the target DNA strand occurs through the action of the cellular DNA repair mechanism [156]. Cells use DSBs, NHEJ, HR, and single-stranded annealing (SSA) during the repair process [156]. The NHEJ re-ligates two DNA ends without requiring a homologous template, by introducing short deletions at the break site. If there is involvement of the DSB repair mechanism, homology-directed repair (HDR) mechanism occurs using the oligonucleotide acts as a template. The introduction of oligonucleotides into the cell during the gene editing process leads to DNA damage. There is a possibility of cellular response to DNA transfection as well. The double-stranded DNA of plasmids that are used during the process induce the expression of genes that are involved in the DNA damage and repair system [156]. ODM has been used for editing several plant genes. The Acetolactate synthase (ALS) gene, which is involved in the first step of the biosynthesis of the branched-chain amino acids leucine, isoleucine, and valine, and in imidazolinone tolerance, was discovered in plants by mutagenesis [157]. Although zinc finger nucleases and other editing tools show great promise in genome editing, oligonucleotide-directed mutagenesis is very effective where simple genetic changes such as insertions, deletions or substitutions are required. In addition, the production and delivery of ODM are very simple and do not require expression of foreign proteins in the cell. This minimizes off-target effects and does not disrupt non-target genes by non-specific integration of genetic material. Although ODM technology is frequently used in plants, the correction rates are comparatively low and the editing largely depends upon the introduced oligonucleotide.

## 11. Cisgenesis and Intragenesis

Genetic mutation and transgene generation are the main techniques used to edit a gene or a genome. Somatic hybridization facilitates the fusion of genetic content from two species that are separated by genetic barriers. The development of genome sequencing technologies has led to the isolation of genes from crossable species; these genes are commonly known as cisgenes. Cisgenesis/intragenesis refers to the transfer of genes or DNA between organisms of the same species, or genetically cross-compatible species, without any accompanying linkage drag [158,159,160]. In cisgenesis, the gene remains unchanged, whereas in intragenesis, a part of the gene (promoter or regulator) is transferred. Cisgenesis may result in a new organism that might not be distinguishable by one obtained from conventional crossbreeding, whereas intragenesis results in an organism that cannot be obtained by conventional crossbreeding [159]. Large-scale cisgenes have been isolated by advanced sequencing approaches; these genes do not carry any marker genes with them. This leads to the modification of the genome, while remaining within the gene pool. Cisgenic plants are more publicly acceptable than transgenic plants. Cisgenesis has been successfully applied in cereal plants where the phytase gene was introduced to confer bioavailability of phosphate [161]. The 1D × 5 and 1Dy10 glutein subunits with native endosperm promoter and terminator were transferred to durum wheat, using a transformation approach to enhance the bread-making property of the wheat [162]. In poplar, five cisgenes (*PtGA20ox7*, *PtGA2ox2*, *Pt RGL1_1*, *PtRGL1_2,* and *PtGAI1*) that are involved in gibberellin signaling were transferred to *Populustremula x alba* [163]. Each cisgene has 1–2 kb of 5′- and 1 kb of 3′-flanking DNA [163]. The *PtGA20ox7* cisgene increased the rate of shoot generation and early growth [163]. The *PtRGL1_2* cisgene resulted in longer xylem fibers, whereas the *PtGAI1* cisgene showed an increased rate of regeneration [163]. The parts of the cisgenes, including promoters and terminators were transferred, into *Populustremula x alba* genome [163]. The cisgenesis and transgenesis approaches use the same genetic modification techniques, except for the introduction of genes from the same plant or from a crossable species whose genes can be transferred by breeding techniques as well. Therefore, cisgenesis is not different from natural breeding programs, and, hence, cisgenesis is safe to use, like traditionally bred plants.

## 12. Plastid Genome and Synthetic Genomics

Approximately 4500 nuclear genes of *A. thaliana* originated from cyanobacteria, and constitute approximately 18% of the gene content of nuclear genome [164]. The transfer of genes from plastids to nuclei is an ongoing process, and shrinkage of the plastid genome will continue to occur in the future. Due to selective pressure, the promoters and intergenic spacer sequences (3′ and 5′ untranslated region) are also becoming smaller. The plastid of seed plant *Cytinus hypocystis* contains the smallest genome (19.4 kb, NC_031150) (Supplementary Figure S1), whereas *Pelargonium transvaalense* contain the largest genome (242.575 kb, NC_031206) (Supplementary Figure S2). If we will consider the plastid genome of green algae, *Ostreococcus tauri* OTTH0595 possesses the smallest genome (71.66 kb, NC_008289) (Supplementary Figure S3), whereas *Floydiella terrestris* possesses the largest genome (521.168 kb, NC_014346) (Supplementary Figure S4), with diverse numbers of genes. Although the genetic map of the plastid genome is circular, it seems quite heterogenous in vivo, and up to 1000 copy numbers of plastid genomes can be found. In addition to the circular genome, multimeric and linear plastid genomes have been reported; these genomes presumably resulted from the homologous recombination of genomic copies [165,166,167]. These diverse sizes of plastid genome can provide valuable importance for genome editing in plants.

Plastids are primarily involved in photosynthesis. Therefore, it is easily speculated that a majority of the genomic content in plastid genomes was utilized for photosynthesis [168,169]. In addition to photosynthetic genes, plastid genomes also contain genes coding for ribosomal RNA (rRNA) and transfer RNA (tRNA), subunit of plastidial ribosomes, intron splicing factor, Clp protease, bacterial-type plastid RNA polymerase (PEP), acetyl-CoA carboxylase, and malonyl-CoA. Plastid genomes encode two different polymerases, i.e., bacterial-type RNA polymerase and bacteriophage-type RNA polymerase, and these polymerases transcribe overlapping sets of genes. The biosynthesis of proteins in plastids occurs with the help of bacterial-type 70S ribosomes [170]. The gene expression event in plastids is highly regulated and occurs in response to developmental and environmental cues [171,172,173]. Considerable numbers of protein products encoded by plastid genomes combine with other proteins to form multi-protein complexes. These complexes consist of nucleus- and plastid-encoded subunits that are involved in the tight coordination of gene expression in plastids and nuclei through anterograde and retrograde signaling pathways [174]. 

Plastid genomes provide a platform for synthetic biological research. Given the smaller genome size, plastid genomes can be manipulated very easily. Synthetic biological research using plastid genomes has been well established in the model organisms *Chlamydomonas reinhardtii* and *Nicotiana tabacum* [175,176]. The presence of homologous recombination in plastids facilitates targeted integration and precise excision, which allows the use of homologous recombination as a suitable tool for genome editing. Although the plastid genome is very compact, one-third of its genomic region is occupied by intergenic regions [168,170]. The intergenic region contains regulatory elements, such as promoters and 5′ and 3′untranslated regions (UTRs), and the presence of truly non-coding regions is negligible [170]. Homologous recombination in plastids leads to the simultaneous modification of two separate regions of the plastid genome by co-transformation, where two or more plasmid vectors can be injected using a biollistic particle gun bombardment approach [170,177]. The co-transformation event can be simultaneously used to transform nuclear genomes as well. The toolkit for plastid genome engineering contains promoters, reporter genes, selectable markers, and 5′ and 3′ UTRs. The biollistic method can allow the transformation of more than 50 kb sequences at a time, and the capacity to incorporate enormous quantities of foreign genomic content makes it an attractive tool for genome editing. These tools allow the use of the smallest-sized genome that contains the smallest possible genomic content that life can accommodate. 

## 13. Conclusions and Future Perspectives

ZFNs and DSBs can potentially be used for precise genome editing in plants, and can have a huge impact in functional genomics studies. This can be very helpful for novel trait discovery in plants, and will be very beneficial when used for improving crops for commercial purposes. Targeted-mutation-related breeding methods can use precise genome editing at specific sites, rather than random mutations, and these methods will reduce the possibility of undesired side effects. Genome editing technology has great potential for revolutionizing crop production worldwide. Although ZFNs and TALENs are routinely used in plant research that uses HDR-based mutations, these tools still produce unwanted results and lead to HDR-altered alleles. Additionally, these technologies can potentially be used to create fusion proteins containing domains other than nucleases. TALE array repeats can be used in order to induce epigenetic modifications in specific genomic regions to induce stable and heritable mutations. Lack of sufficient genetic data set to address the sequence specificities for different genome editing tools is the biggest limitation for target prediction, and a larger effort is necessary to address this problem. However, the presence of DNA-binding domain is enough to predict the target to little extent. The CRISPR/Cas9 system can be very useful for post-transcriptional control of gene expression. ABE-mediated genome editing will be very useful for generating point mutations/deletions with high accuracy and less indels. Chromosome engineering and the synthetic plant genome approach can be used as a potential tool for DSB-mediated genome editing in future. These genetic devices can be integrated into the genetic circuit to switch on and off to a particular trait or pathways. Introduction of a “genome editing” database that harbor experimental references, as well as in-silico prediction data of model organisms could be of particular interest. From the database, a researcher could be able to find suitable genome editing tools for a complex gene.

## Supplementary Materials

The following are available online at www.mdpi.com/2073-4425/8/12/399/s1.
